# Close evolutionary relationship between rice black-streaked dwarf virus and southern rice black-streaked dwarf virus based on analysis of their bicistronic RNAs

**DOI:** 10.1186/s12985-019-1163-3

**Published:** 2019-04-27

**Authors:** Zenghui Wang, Chengming Yu, Yuanhao Peng, Chengshi Ding, Qingliang Li, Deya Wang, Xuefeng Yuan

**Affiliations:** 1 College of Life Sciences, Zaozhuang University, Zaozhuang, 277160 People’s Republic of China; 2 College of Plant Protection, Shandong Agricultural University, Tai’an, 271018 People’s Republic of China

**Keywords:** RBSDV, SRBSDV, Phylogenetic analysis, Recombination, Selection pressure

## Abstract

**Background:**

*Rice black-streaked dwarf virus* (RBSDV) and *Southern rice black-streaked dwarf virus* (SRBSDV) seriously interfered in the production of rice and maize in China. These two viruses are members of the genus *Fijivirus* in the family *Reoviridae* and can cause similar dwarf symptoms in rice. Although some studies have reported the phylogenetic analysis on RBSDV or SRBSDV, the evolutionary relationship between these viruses is scarce.

**Methods:**

In this study, we analyzed the evolutionary relationships between RBSDV and SRBSDV based on the data from the analysis of codon usage, RNA recombination and phylogenetic relationship, selection pressure and genetic characteristics of the bicistronic RNAs (S5, S7 and S9).

**Results:**

RBSDV and SRBSDV showed similar patterns of codon preference: open reading frames (ORFs) in S7 and S5 had with higher and lower codon usage bias, respectively. Some isolates from RBSDV and SRBSDV formed a clade in the phylogenetic tree of S7 and S9. In addition, some recombination events in S9 occurred between RBSDV and SRBSDV.

**Conclusions:**

Our results suggest close evolutionary relationships between RBSDV and SRBSDV. Selection pressure, gene flow, and neutrality tests also supported the evolutionary relationships.

**Electronic supplementary material:**

The online version of this article (10.1186/s12985-019-1163-3) contains supplementary material, which is available to authorized users.

## Introduction

In major rice-growing regions, virus diseases occurred frequently and caused severe damages to rice, among which Rice black-streaked dwarf virus (RBSDV) and Southern rice black-streaked dwarf virus (SRBSDV) are major viral pathogens in China. RBSDV is a member of the genus *Fijivirus* in the family Reoviridae, which infects rice, maize and wheat [1–3]. RBSDV-infected rice plants have typical dwarf symptoms, such as dark leaves and small white waxy galls on the underside of the leaves [2, 3]. Another disease with analogous symptoms was first reported in Guangdong Province, China in 2001 [4], and later was identified as a disease caused by *Southern rice black-streaked dwarf virus* (SRBSDV), a new member of the genus *Fijivirus* [5]. SRBSDV was subsequently reported to be found throughout southern China and Vietnam [6]. In recent years, both viruses caused a significant reduction in rice yield. However, the identification of these diseases simply based on the symptoms on rice leaves is problematic.

**Fig. 1:**
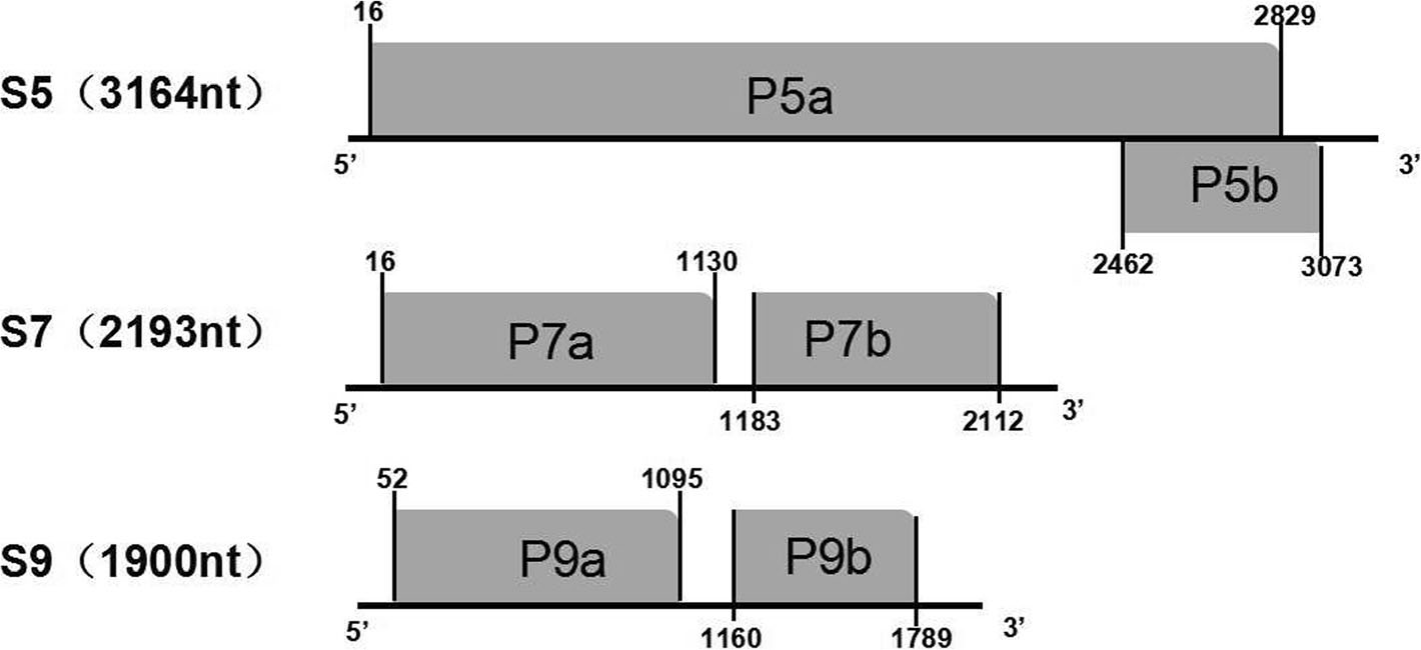
Genome organization of S5, S7 and S9. The dark area is ORF region

RBSDV and SRBSDV are effectively transmitted by the small brown planthopper (SBPH, *Laodelphax Srtiatellus* Fallen) and the white-backed planthopper (WBPH, *Sogatella furcifera* Horvath), respectively in a persistent propagative manner [5–7]. RBSDV and SRBSDV have similar genome organizations with 10 dsRNA segments that totally encode 13 proteins [1, 5, 8]. Among the 10 dsRNA segments, the dsRNA S5, S7 and S9 are comprised of two open reading frames (ORFs) that encode for two proteins. It is suggested that the expression of the second protein encoded by the bicistronic RNAs is strictly regulated. P7–1 can cause male sterility due to non-dehiscent anthers in Arabidopsis [9]. P7–2 can interact with SKP1, a core subunit of SCF ubiquitin ligase [10]. P9–1 may participate in the processes of viroplasma nucleation and virus morphogenesis through the recruitment of P6 [11]. Although some studies have reported the phylogenetic analysis on RBSDV or SRBSDV [12–14], the evolutionary relationship between RBSDV and SRBSDV is scarce. In this study, we accessed the evolutionary relationship between RBSDV and SRBSDV using on the data the analysis of codon usage, RNA recombination and phylogenetic relationship, selection pressure and genetic characteristic of bicistronic RNAs (S5, S7 and S9) (Fig. 1). By analyzing the bicistronic RNAs (S5, S7 and S9) in RBSDV and SRBSDV, we identified the evolutionary relationships between the viruses, and potentially discovered some information of gene expression and regulation. (For the abbreviations, refer to Table 1).

**Table 1:** List of abbreviations

Abbreviations	Unabbreviated Form
RBSDV	Rice black-streaked dwarf virus
SRBSDV	Southern rice black-streaked dwarf virus
ORFs	Open reading frames
ENC	Effective number of codons
CBI	Codon bias index
GC3_S_	G + C content at third position
GC_C_	G + C content at coding positions
SBPH	Small brown planthopper
WBPH	White-backed planthopper
*RSCU*	Relative synonymous codon usage
RDP4	Recombination Detection Program version

## Results

### Analysis of codon usage bias based on ORFs of S5, S7 and S9 in RBSDV and SRBSDV

Using DnaSP 5.0, codon usage bias analysis was performed on ORFs of S5, S7 and S9 in both RBSDV and SRBSDV. When the gene was highly expressed, the gene had high codon bias with the low effective number of codons (ENC) value. Data from RBSDV and SRBSDV showed that ORFs in S5 had higher ENC, and ORFs in S7 had lower ENC (Table 2). For RBSDV, ORF7–2 had the lowest ENC (39.958), and ORF5–2 had the highest ENC (55.334). For SRBSDV, ORF7–1 has lowest ENC (41.394) and ORF5–1 had highest ENC (51.455) (Table 1). Codon bias index (CBI) showed the pattern of negative correlation with ENC. Both ENC and CBI suggested that ORFs in S7 of RBSDV and SRBSDV has relatively higher codon usage bias, while ORFs in S5 of RBSDV and SRBSDV had relatively lower codon usage bias (Table 2). In addition, data for GC3_S_ and GC_C_ had shown that G + C content was low in all analyzed ORFs.

**Table 2:** Codon usage for ORFs of S5, S7 and S9 in Rice black-streaked dwarf virus and Southern rice black-streaked dwarf virus

Virus	ORFs	ENC	CBI	GC3_S_	GC_C_
RBSDV	ORF5–1	49.982	0.323	0.329	0.372
	ORF5–2	55.334	0.312	0.384	0.378
	ORF7–1	45.780	0.498	0.231	0.354
	ORF7–2	39.958	0.598	0.227	0.324
	ORF9–1	47.382	0.422	0.298	0.356
	ORF9–2	43.354	0.541	0.189	0.296
SRBSDV	ORF5–1	51.455	0.323	0.365	0.379
	ORF5–2	46.595	0.342	0.334	0.360
	ORF7–1	41.394	0.569	0.210	0.343
	ORF7–2	43.882	0.539	0.217	0.304
	ORF9–1	45.992	0.445	0.291	0.356
	ORF9–2	47.494	0.516	0.256	0.322

**Fig. 2:**
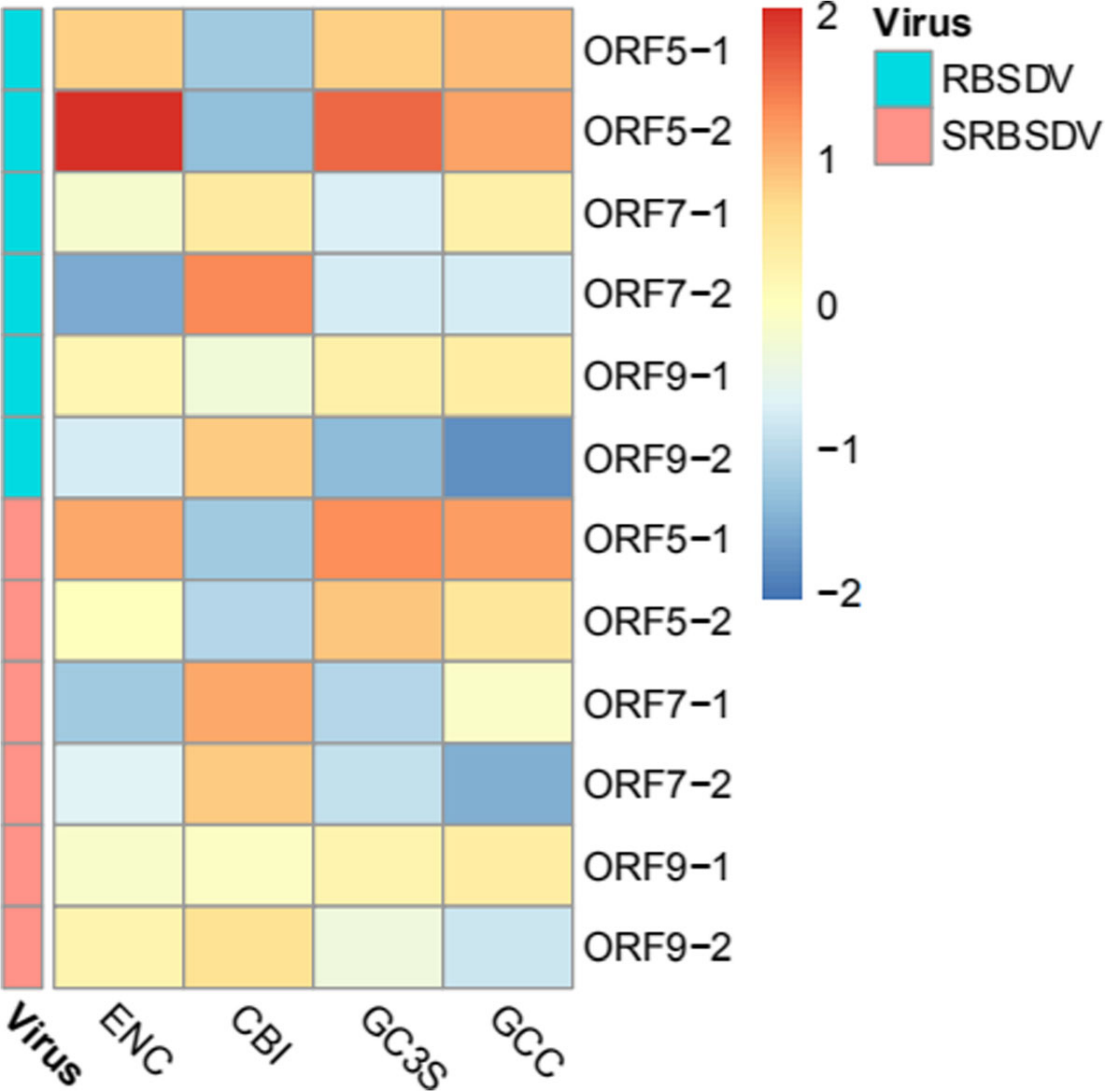
A heat map of the codon usage in the coding region of the bicistronic RNAs (S5, S7 and S9)

For determining the optimal codons used in all ORFs, the average relative synonymous codon usage (*RSCU)* value was determined (Additional file 1: Table S1). GUA (V) in S7–1, GAA (E) /GAG (E) in S7–2 of SRBSDV as well as GGC (G)/ GGG (G) in S5–2 of RBSDV, and UGG in ORFs of S5/S7 in RBSDV and SRBSDV showed no codon usage bias, except AUG (M) (Additional file 1: Table S1). In addition, the majority of optimal codons with RSCU values approximately equal to 1 end with U or A, indicating that the codon usage in RBSDV and SRBSDV was biased towards synonymous codons. In addition,we produced a graphical heat map for showing the codon usage in the coding region (Fig. 2).

### Recombination analysis

Recombination analysis, used to identify potential evolutionary relationships between RBSDV and SRBSDV, detected no recombination for S5. Only one recombination was detected for S7 (Table 3). All of the recombination sequences, major and minor parents were the sequences from RBSDV S7. For S9, we detected higher recombination frequency with a total of 9 recombination events (Table 3). We also detected recombination events between RBSDV S9 and SRBSDV S9. For event 9–7, recombination of position 82–1184 in RBSDV S9 (HQ731500) was derived from major parent SRBSDV S9(HM998852) and minor parent SRBSDV S9 (HF955003). For other events, drivers were RBSDV S9 and minor parents were SRBSDV S9. These results suggest that S9 of RBSDV and SRBSDV underwent frequent RNA recombination during natural evolution. It also suggests that genome information of RBSDV and SRBSDV may be exchanged when infecting the same host plant.

**Table 3:** Recombination events of S7 and S9 in RBSDV and SRBSDV

Event number	Recombination sequence	Breakpoint		Supporting software	Major Parent	Minor Parent	P-value
		Begin	End				
7–1	HF955011(S7)	1262	1313	RGMCS	HF954991	AY147039	1.456 × 10^−9^
9–1	AF459812(S9)	16	1472	RGMS	HQ7315000	HF955003	2.390 × 10^−10^
9–2	AF536564(S9)	16	1472	RGMS	HQ7315000	Unknown (HF955003)	2.390 × 10^−10^
9–3	AJ291706(S9)	4	1472	RGMS	HQ7315000	Unknown (HF955003)	1.081 × 10^− 62^
9–4	AY039705(S9)	16	1469	RGMS	HQ731500	Unknown (HF955003)	4.518 × 10^−21^
9–5	AY050487(S9)	16	1469	RGMS	HQ731500	Unknown (HF955003)	1.081 × 10^−62^
9–6	HF955013(S9)	16	1469	RGMCS	HQ731500	Unknown (HF955003)	2.673 × 10^−9^
9–7	HQ731500(S9)	82	1184	RGBMS	Unknown (HM998852)	HF955003	1.011 × 10^−14^
9–8	KC134297(S9)	4	1472	RGMS	HQ731500	Unknown (HF955003)	7.524 × 10^−14^
9–9	KM921681(S9)	4	1469	RGBMS	HQ731500	Unknown (HF955003)	4.625 × 10^−19^

Note: R, RDP; G, GENECONV; M, MAXCHI; C, CHIMAERA; S, SISCAN; B, BOOTSCAN

### Phylogenetic analysis of S5, S7 and S9 in RBSDV and SRBSDV

**Fig. 3:**
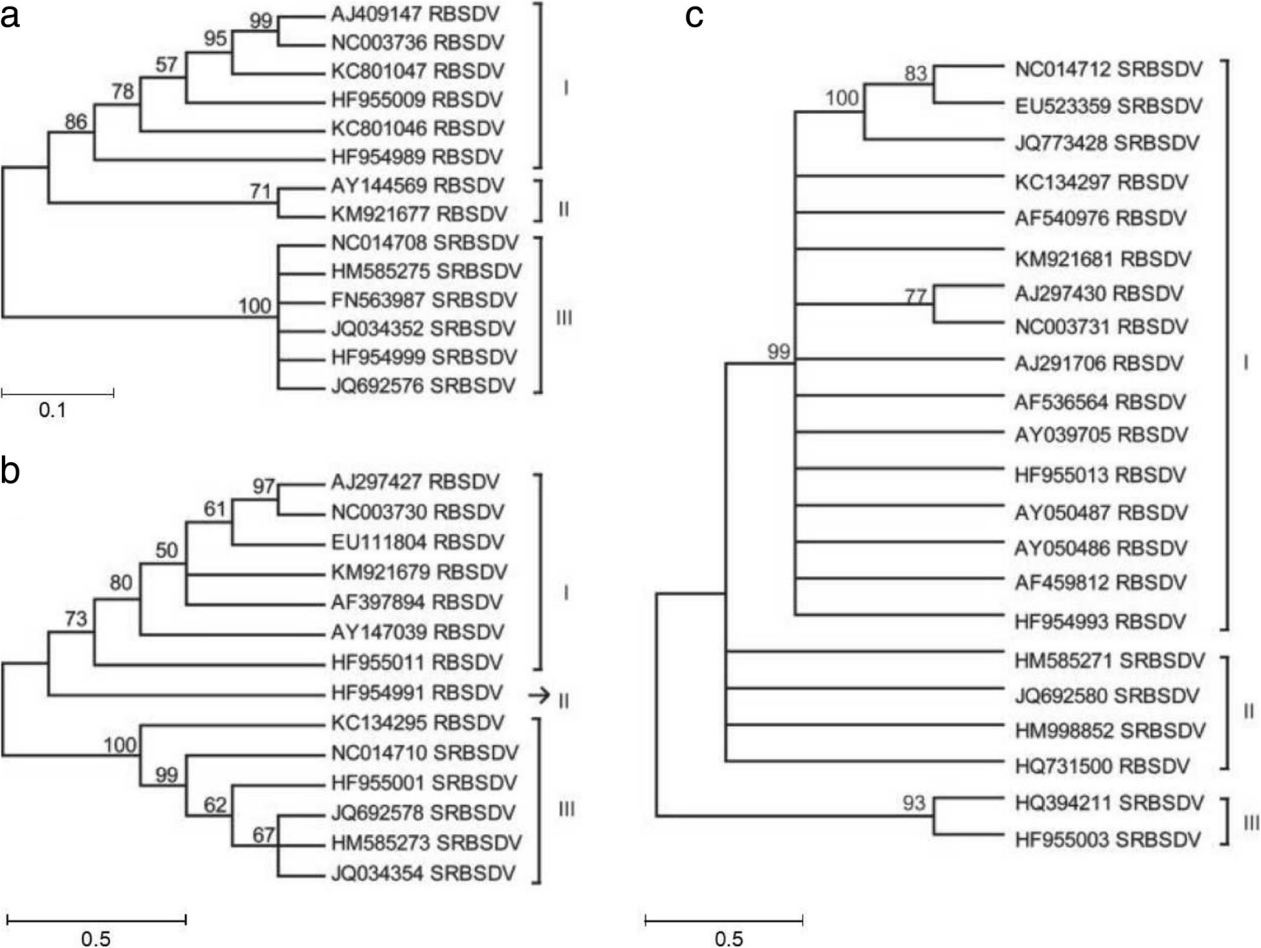
Phylogenetic trees based on S5, S7 and S9 of RBSDV and SRBSDV. **a**: Phylogenetic tree of S5; **b**: Phylogenetic tree of S7; **c**: Phylogenetic tree of S9

For further identifying the evolutionary relationship between RBSDV and SRBSDV, phylogenetic trees were constructed based on S5, S7 and S9 via MEGA 5.0, respectively (Fig. 3). In a phylogenetic tree of S5, RBSDV S5 formed two clades (I and II) and SRBSDV S5 formed clade III (Fig. 3a). It implies S5 independently evolved in RBSDV and SRBSDV. In phylogenetic tree of S7, there are three clades. Clade I and II consist of RBSDV S7. Clade III includes RBSDV S7 (KC134295) and SRBSDV S7 (Fig. 3b). These results highlight a close relationship between RBSDV S7 and SRBSDV S7. Phylogenetic tree of S9 also produced three clades with clade I consists of three SRBSDV S9 s and thirteen RBSDV S9 s, clade II includes two SRBSDV S9 s and two RBSDV S9 s and clade III contains two SRBSDV S9 s (Fig. 3c). These results a potential genetic exchange between RBSDV S9 and SRBSDV S9 during natural evolution.

### Selection pressure on ORFs of S5, S7 and S9

The selection pressure on S5, S7 and S9 in RBSDV and SRBSDV were measured by calculating the ratios of non-synonymous (*dN*) and synonymous sites (*dS*). When the value of ratio is more than 1, the gene is considered to be under positive or diversifying selection; when the value of ratio is less than 1, the selection is negative or purifying selection; and when the ratio is equal to 1, the selection is neutral. The statistics were performed on different subpopulations based on host and virus types (Table 4). Based on *dN*/*dS* value, ORF7–2 in viruses infecting maize underwent neutral selection with *dN*/*dS* ratio equal to 1, while other ORFs in different subpopulations underwent negative selection with *dN*/*dS* ratio less than 1 (Table 4). Different ratios indicate that ORFs experienced different negative selection pressure.

**Table 4:** Non-synonymous and synonymous substitution in ORFs of S5, S7 and S9

	ORFs	*dN*	*dS*	*dN*/ *dS*		ORFs	*dN*	*dS*	*dN*/ *dS*
RBSDV	ORF5–1	0.013 ± 0.002	0.105 ± 0.008	0.124	SRBSDV	ORF5–1	0.006 ± 0.001	0.021 ± 0.003	0.286
	ORF5–2	0.015 ± 0.004	0.053 ± 0.012	0.283		ORF5–2	0.005 ± 0.002	0.012 ± 0.006	0.417
	ORF7–1	0.002 ± 0.001	0.071 ± 0.009	0.028		ORF7–1	0.001 ± 0.001	0.009 ± 0.004	0.111
	ORF7–2	0.010 ± 0.002	0.089 ± 0.013	0.112		ORF7–2	0.001 ± 0.001	0.012 ± 0.006	0.083
	ORF9–1	0.033 ± 0.004	0.259 ± 0.028	0.127		ORF9–1	0.008 ± 0.002	0.014 ± 0.003	0.571
	ORF9–2	0.032 ± 0.012	0.348 ± 0.101	0.092		ORF9–2	0.090 ± 0.011	0.193 ± 0.049	0.466
Maize	ORF5–1	0.002 ± 0.001	0.025 ± 0.006	0.08	Rice	ORF5–1	0.605 ± 0.067	1.039 ± 0.135	0.582
	ORF5–2	0.004 ± 0.003	0.028 ± 0.015	0.143		ORF5–2	1.289 ± 0.391	1.363 ± 0.362	0.946
	ORF7–1	0.001 ± 0.001	0.023 ± 0.007	0.043		ORF7–1	0.085 ± 0.012	0.772 ± 0.140	0.110
	ORF7–2	0.003 ± 0.002	0.003 ± 0.011	1.0		ORF7–2	0.168 ± 0.055	0.994 ± 0.316	0.169
	ORF9–1	0.101 ± 0.010	0.763 ± 0.113	0.132		ORF9–1	0.091 ± 0.010	0.683 ± 0093	0.133
	ORF9–2	0.102 ± 0.046	1.081 ± 0.394	0.094		ORF9–2	0.122 ± 0.030	0.980 ± 0.280	0.124

Based on *dN* and *dS* value, non-synonymous and synonymous substitution rates in ORFs were also identified. ORF5–1 and ORF5–2 in viruses infecting rice have relatively high *dN* value (0.605 and 1.289, respectively), indicating the high rates of non-synonymous substitutions in these ORFs (Table 3). *dS* values of ORF9–1 (0.259) and ORF9–2 (0.348) in RBSDV and ORF9–2 (0.193) in SRBSDV were relatively high, indicating frequent synonymous nucleotide substitutions in these ORFs (Table 4). For maize infecting viruses, ORF9–1 and ORF9–2 had relatively high *dS* value (0.763 and 1.081, respectively). However, for rice infecting viruses, all ORFs, especially ORF5–1 (*dS*: 1.039) and ORF5–2 (*dS*: 1.363), have relatively high *dS* values with ORF5–1 (*dS*: 1.039) and ORF5–2 (*dS*: 1.363) had the highest values. It suggests that RBSDV and SRBSDV have a higher synonymous substitution rate when infecting rice, rather than maize.

### Genetic differentiation and gene flow in S5, S7 and S9

Genetic differentiation and gene flow were analyzed using DnaSP 5.0. Test statistics Ks*, Z, and Snn (see methods) were used to access genetic differentiation in S5, S7 and S9; and *Fst* and *Nm* were used to measure gene flow*.* The statistics were performed on different subpopulations according to virus type, host type and region (Table 5). The value of Ks* ranged from 0 to 6.06298 (Table 5), which indicated the variations between the sequences (Table 5). Compared to several kb genomes, the genetic difference was relatively low. The highest genetic difference (Ks*: 6.06298) was in S9 of SRBSDV, but there was no genetic difference in S5, S7 or S9 (Ks*: 0) between Zhejiang and Anhui (Table 5).

**Table 5:** Genetic differentiation and gene flow between subpopulations

sequences	subpopulations	Ks*	*Z*	Snn	*Nm*	*Fst*
S5	RBSDV	4.68574	2.63348	0.06250	5.08	−0.21377
	SRBSDV	3.52466	1.99848	0.16667	1.40	−0.10526
	RBSDV-SRBSDV	4.03210	2.77618	1.0000	0.02	0.96173
	Maize-Rice	3.82262	7.93333	1.0000	4.56	0.78107
	Jiangsu-Zhejiang	4.75212	7.83333	0.83333	0.71	0.40672
	Zhejiang-Anhui	0.0000	0.00000	0.75000	0.50	0.42143
	Jiangsu-Anhui	4.75212	1.83501	0.33333	12.0	0.13397
S7	RBSDV	4.52336	2.77535	0.16667	1.52	−0.01867
	SRBSDV	1.90126	1.45543	0.2000	0.99	−0.18605
	RBSDV-SRBSDV	3.70347	2.79015	0.92857	0.09	0.86866
	Maize-Rice	3.54044	1.95144	0.85714	5.20	0.14586
	Jiangsu-Zhejiang	3.04377	1.13373	1.0000	0.61	0.57143
	Zhejiang-Anhui	0.0000	0.0000	0.75000	0.50	0.61463
	Jiangsu-Anhui	3.04377	0.59725	0.80000	30.0	0.48168
S9	RBSDV	3.89158	3.54343	0.35714	1.75	0.04670
	SRBSDV	6.06298	2.36085	0.62500	2.80	−0.13159
	RBSDV-SRBSDV	4.85276	4.08636	0.73810	0.62	0.33722
	Maize-Rice	3.79931	2.70675	0.9000	8.51	0.02095
	Jiangsu-Zhejiang	2.55985	1.66296	0.71429	4.62	0.00696
	Zhejiang-Anhui	0.00000	0.00000	0.71429	0.61	−0.00378
	Jiangsu- Anhui	2.55985	1.85037	0.25000	4.62	−0.10000

Different subpopulations showed different gene flow. For S5, gene flow within RBSDV or SRBSDV was frequent with the absolute value of *Fst* less than 0.33 (Table 5). This pattern also applied to S7 and S9 within RBSDV and SRBSDV. For S5, S7 and S9 between RBSDV and SRBSDV, there was an infrequent gene flow with *Fst* more than 0.33. We also observed a substantial local differentiation with *Nm* less than 1. For S5 and S7 between maize and rice, there was an infrequent gene flow with *Fst* more than 0.33. However, we found a low frequency of genetic differentiation with *Nm* more than 1. For S9 between maize and rice, there is a frequent gene flow with *Fst* less than 0.33 and low frequency of genetic differentiation with *Nm* more than 1. Different regions had different gene flow patterns. For S5 and S7 between Jiangsu and Zhejiang, or between Zhejiang and Anhui, there is an infrequent gene flow with substantial local differences. For S5, S7 and S9 between Jiangsu and Anhui, there was a low frequency of genetic differentiation with *Nm* more than 1.

### Neutrality tests on S5, S7 and S9 in RBSDV and SRBSDV

The neutrality test was based on the differences between the number of segregating sites and the average number of nucleotide differences. To testing the neutrality hypothesis and perform population demography, values for Tajima’s D, Fu and Li’s D, and Fu and Li’s F were calculated using DnaSP version 5.0 (Table 6). Except for the Tajima’s D value of ORF7–2 in RBSDV, all Tajima’s D values of other ORFs in RBSDV and SRBSDV are significantly far away from 0, which indicates these ORFs were under natural selection.

**Table 6:** Neutrality test of S5, S7 and S9 in RBSDV and SRBSDV

RBSDV	Tajima’s D	Fu & Li’s D	Fu & Li’s F	SRBSDV	Tajima’s D	Fu & Li’s D	Fu &Li’s F
ORF5–1	− 0.00508	0.40941	0.34886	ORF5–1	−0.66188	− 0.61629	−0.68738
ORF5–2	−0.06959	0.36868	0.29669	ORF5–2	−0.79480	−0.85062	− 0.90575
ORF7–1	−1.53102	−1.60532	−1.78365	ORF7–1	− 0.07339	−0.07339	− 0.07686
ORF7–2	− 0.24789	0.69109	0.52040	ORF7–2	−0.80734	− 0.80734	−0.84548
ORF9–1	−2.01232	−2.30495	−2.55773	ORF9–1	−1.26116	−1.05424	− 1.23976
ORF9–2	−2.04066	−2.35516	−2.60827	ORF9–2	−2.03684	−2.31023	−2.52287

In terms of Fu & Li’s D and F statistical tests, we found different patterns exist in RBSDV and SRBSDV. For SRBSDV, all values of Fu & Li’s D and F are negative, indicating that S5, S7 and S9 of SRBSDV have a tendency to expand with a low frequency of polymorphism. RBSDV, ORF7–1, ORF9–1 and ORF9–2 had a low frequency of polymorphism with a negative value of Fu & Li’s D and F. In contrast ORF5–1, ORF5–2 and ORF7–2 may have a high frequency of polymorphism with a positive value of Fu & Li’s D and F.For SRBSDV, all values of Fu and Li’s D and F were negative, indicating that S5, S7 and S9 of SRBSDV may have a tendency to expand with a low frequency of polymorphism.

## Discussion

Consistent with increasing trends in global export of agriculture commodities, RBSDV and SRBSDV have been spreading rapidly worldwide. During a rapid spread phase, these viruses may have maintained different evolutionary trajectories based on specific host and the environment. Although some previous studies have reported the phylogenetic analyses on RBSDV or SRBSDV separately [12–14], but the studies on the evolutionary relationship between RBSDV and SRBSDV was rare. In this study, we examined a potential evolutionary relationship between these viruses by performing an evolutionary analysis of S5, S7 and S9 in RBSDV and SRBSDV.

Codon usage bias has been reported for viruses (CITATION), including those infect humans (CITATION). Such biases provide information about virus-host coevolution [15, 16]. A detailed comparative analysis was performed to evaluate the degrees of codon usage bias about ORFs of S5, S7 and S9. In general, RBSDV and SRBSDV showed similar patterns of codon usage bias for ORFs of S5, S7 and S9 (Table 1). Compared with other ORFs, ORFs in S7 of RBSDV and SRBSDV had relatively higher codon usage bias with lowest ENC value. Low ENC value was usually correlated to high expression [17]. The correlation implies the evolutionary requirements for high expression of the ORFs, particularly ORF7–2, in S7. This potential high expression of ORF7–2 was supported by a study of that evaluated a intergenic region between ORF7–1 and ORF7–2 that contains a high activity IRES (Yuan et al., in preparation). In addition, 5’UTR in S3 or S10 of RBSDV enhanced the translation of FLuc reporter gene and possess IRES activity in the absence or presence of the 5’cap structure. To the best of our knowledge, this is the first report on the effect of untranslated regions of ds RNA viruses on translation [18].

Recombination is a major evolutionary mechanism that commonly found in many plant RNA viruses, including RBSDV and SRBSDV [12, 13, 19, 20]. Such recombination may prevent accumulation of mutations, help adapt to new hosts or environmental changes and overcome host resistance [21–23]. In this study, recombination events were detected between RBSDV and SRBSDV (e.g., recombination event 9–7) (Table 3). These results highlight the potential evolutionary relationship between RBSDV and SRBSDV. Moreover, phylogenetic analysis supported a evolutionary relationship. Clade III in the phylogenetic tree of S7 contained both RBSDV S7 and SRBSDV S7. Similar pattern was also found in clade I and II in the phylogenetic tree of S7. The close evolutionary relationship highlights the frequent genetic exchange between RBSDV and SRBSDV when they infected the same host. It also suggests that these viruses may evolved from a single ancestor during evolution. Although the high nucleotide identity of these RBSDV and SRBSDV isolates, they may take different evolutionary path, and showed different host range or pathogenicity in the field. Finally, the selection pressure, gene flow, and recombination together promoted the evolution of the RBSDV and SRBSDV. The evolutionary trends under natural conditions may have a directing significance to the prevention and control of viral diseases.

## Conclusions

RBSDV and SRBSDV presented similar patterns of codon usage bias: ORFs in S7 with higher codon usage bias and ORFs in S5 with lower codon usage bias. Some isolates from RBSDV and SRBSDV formed a clade in the phylogenetic tree of S7 and S9. In addition, some recombination events in S9 occurred between RBSDV and SRBSDV. Our results implied a close evolutionary relationship between RBSDV and SRBSDV. Analysis of selection pressure, gene flow, and neutrality test also supports this potential relationship.

## Materials and methods

### Sources of virus sequences

The sequences of bicistronic RNAs (S5, S7 and S9) in RBSDV and SRBSDV were referenced from http://www.ncbi.nlm.nih.gov/nucleotide/.

### Codon usage bias analysis

Codon usage bias analysis was performed using the DnaSP 5.0 [24–26]. An effective number of codons (ENC) indicates a bias for synonymous codons rather than codon number or amino acid composition [26, 27]. The value of ENC ranges from 20 to 61 [17]. ENC value 20 indicates that only one type of codon is used for each amino acid and the codon bias is maximum; When the value is 61, all synonymous codons of each amino acid are equally used and there was no codon bias [26]. When the gene is highly expressed, it has high codon bias with low ENC value [17]. The codon bias index (CBI) is a measure of the deviation from the equal use of synonymous codons. The value of CBI ranges from 0 to 1 [28]. If the value of CBI is higher, it indicates that the codon bias is higher. G + C3s is the G + C content at the third position. G + Cc is G + C content at coding positions [26]. Relative Synonymous Codon Usage (*RSCU*) values represent the number of times a particular codon is observed, relative to the number of times that the codon would be observed for a uniform synonymous codon usage. In the absence of any codon usage bias, the *RSCU* values would be 1.00. A value less than 1 (or more than 1) indicates that the codons are used less frequently (or more frequently) than expected [24].

### Recombination and phylogenetic analysis

Recombination analysis was performed using RDP, GENECONV, BOOTSCAN, MAXCHI, CHIMAERA, 3SEQ and SISCAN programs in RDP 4 software package with a default setting [28]. When the *p*-values was less than 10^− 6^ and the value of Z was more than 3 at the same time, the events supported by at least four programs were considered to be recombination [19, 29, 30]. Phylogenetic analysis was performed using MAGE5. Phylogenetic trees were constructed using the neighbor-joining (NJ) method as described previously [31]. The number of bootstrap replicates was 1000. Branches with less than 50% bootstrap value were collapsed.

### Detection of selection pressure

Selection pressure was performed using the software MAGE5.0 as described in previous studies [31–33] The ratios of *dN*/*dS* was used to describe the selection pressure. Here, *dN* is the average number of non-synonymous substitutions per site. *dS* is the average number of synonymous substitutions per site [31]. When a value of *dN*/*dS* is more than 1, the gene is considered to be under positive or diversifying selection; when a value of *dN*/*dS* is less than 1, the selection is negative or purifying; Finally, when *dN*/*dS* is equal to 1, the selection is neutral [31, 32].

### Analysis of genetic differentiation and gene flow

Genetic differentiation and gene flow analyses were performed using the software DnaSP 5.0 [34]. Genetic differentiation was evaluated using sequence based statistics, Ks*, Z, and Snn (the nearest-neighbor statistic). where Ks* is a weighted average of differences between the sequences. Z is a rank statistic, and Snn represents how often the nearest neighbors’ in the sequences are from the same location [35, 36]. Gene flow was estimated by measuring *Fst* and *Nm,* where *Fst* represents the component of genetic variation between populations and *Nm* represents the female effective size of female population (N) and migration rate among populations (m) [34]. The *Fst* values ranges from 0 to 1. When a value is more than 0.33, it implies that there is an infrequent gene flow. A value of *<* 0.33 implies a frequent gene flow [20, 34]. A *Nm* value of less than 1, it implies a genetic drift with a substantial local differentiation. A value of *Nm* is more than 1 implies a frequent gene flow with a low frequency of genetic differentiation [34].

### Neutrality tests and population demography

Software DnaSP5.0 was used to detect the value of Tajima’s D, Fu & Li’s D and F statistics [34]. The Tajima’s D test statistic was proposed for testing the hypothesis that states all the mutations are selectively neutral [37]. The test compares the differences between the number of segregating sites and the average number of nucleotide differences. A Tajima’s D value away from 0(i.e. < or > 0) implies population under natural selection. The Fu & Li’s D test statistics measure the differences between the number of singletons and the total number of mutations [38]. The F test statistics measures the differences between the number of singletons and the average number of nucleotide differences between pairs of sequences [38, 39]. A negative value for Fu & Li’s D and F implies a low population diversity but still tends to expand. A negative value further implies a population with a low frequency of polymorphism.

## Additional file

Additional file 1: **Table S1.** Relative synonymous codon usage (RSCU) values for each codon in S5, S7 and S9 of RBSDV and SRBSDV. **Table S2.** The sequence of RBSDV and SRBSDV information used in this paper. (DOCX 29 kb)
