# Supplementary material for: Close evolutionary relationship between rice black-streaked dwarf virus and southern rice black-streaked dwarf virus based on analysis of their bicistronic RNAs
Source: Virol J. 2019 Apr 27;16:53. doi: 10.1186/s12985-019-1163-3 (PMC6486993; doi:10.1186/s12985-019-1163-3)
Supplement: Supplementary file 1 — Table S1. Relative synonymous codon usage (RSCU) values for each codon in S5, S7 and S9 of RBSDV and SRBSDV. Table S2. The sequence of RBSDV and SRBSDV information used in this paper. (DOCX 29 kb) [file 12985_2019_1163_MOESM1_ESM.docx]

Table S1. Relative synonymous codon usage(RSCU) values for each codon in S5,S7 and S9 of RBSDV and SRBSDV

| Codon | RBSDV | | | | | | SRBSDV | | | | | |
| --- | --- | --- | --- | --- | --- | --- | --- | --- | --- | --- | --- | --- |
|  | S5-1 | S5-2 | S7-1 | S7-2 | S9-1 | S9-2 | S5-1 | S5-2 | S7-1 | S7-2 | S9-1 | S9-2 |
| UUU(F) | 1.17 | 1.42 | 1.38 | 1.61 | 1.35 | 1.86 | 1.14 | 1.02 | 1.63 | 1.76 | 1.34 | 1.74 |
| UUC(F) | 0.83 | 0.58 | 0.63 | 0.39 | 0.65 | 0.14 | 0.86 | 0.98 | 0.38 | 0.24 | 0.66 | 0.26 |
| UUA(L) | 1.03 | 0.9 | 2.35 | 2.65 | 1.13 | 1.36 | 0.56 | 2.32 | 2.72 | 3.6 | 0.96 | 1.87 |
| UUG(L) | 1.75 | 1.4 | 1.31 | 1.5 | 1.91 | 1.19 | 1.91 | 1.35 | 1.08 | 1.29 | 2.1 | 1.41 |
| CUU(L) | 1.32 | 1.15 | 1.24 | 0.95 | 1.24 | 2.29 | 1.42 | 1.39 | 0.9 | 0.32 | 1.31 | 1.98 |
| CUC(L) | 0.63 | 0.86 | 0.59 | 0.19 | 0.62 | 0.12 | 0.88 | 0 | 0.15 | 0.16 | 0.53 | 0.06 |
| CUA(L) | 0.68 | 1.44 | 0.48 | 0.35 | 0.59 | 0.64 | 0.56 | 0.93 | 0.84 | 0.47 | 0.73 | 0.37 |
| CUG(L) | 0.59 | 0.25 | 0.02 | 0.35 | 0.51 | 0.41 | 0.67 | 0 | 0.31 | 0.16 | 0.37 | 0.3 |
| AUU(I) | 1.83 | 1.28 | 1.67 | 1.46 | 1.64 | 1.99 | 1.87 | 1.13 | 2.05 | 2.29 | 1.76 | 1.96 |
| AUC(I) | 0.7 | 1.06 | 0.81 | 0.63 | 0.91 | 0.55 | 0.9 | 0.94 | 0.27 | 0.14 | 0.81 | 0.62 |
| AUA(I) | 0.46 | 0.66 | 0.52 | 0.91 | 0.45 | 0.46 | 0.23 | 0.94 | 0.68 | 0.57 | 0.42 | 0.42 |
| AUG(M) | 1 | 1 | 1 | 1 | 1 | 1 | 1 | 1 | 1 | 1 | 1 | 1 |
| GUU(V) | 1.87 | 0 | 2.1 | 2.44 | 2.7 | 1.59 | 2.17 | 1.45 | 1.8 | 2 | 2.73 | 1.73 |
| GUC(V) | 0.8 | 1.51 | 0.63 | 0.39 | 0.77 | 0.49 | 0.72 | 1.09 | 0.8 | 0.67 | 0.61 | 0.51 |
| GUA(V) | 0.8 | 1.55 | 1.07 | 0.55 | 0.27 | 1.29 | 0.46 | 0.73 | 1 | 0.89 | 0.42 | 1.19 |
| GUG(V) | 0.52 | 0.93 | 0.2 | 0.62 | 0.27 | 0.63 | 0.64 | 0.73 | 0.4 | 0.44 | 0.24 | 0.57 |
| UCU(S) | 1.42 | 2.38 | 1.79 | 2.41 | 2.61 | 2.05 | 1.45 | 3.53 | 1.82 | 1.81 | 2.37 | 2.06 |
| UCC(S) | 0.8 | 0.44 | 0.45 | 0.05 | 0.48 | 0.12 | 1.06 | 0 | 0.58 | 0.28 | 0.61 | 0.57 |
| UCA(S) | 1.76 | 1.41 | 1.19 | 0.48 | 1.74 | 1.57 | 1.32 | 1.2 | 0.72 | 0.88 | 1.86 | 1.2 |
| UCG(S) | 0.37 | 1.29 | 0.02 | 0.21 | 0.49 | 0.6 | 0.41 | 0.47 | 0.29 | 0.47 | 0.37 | 0.55 |
| CCU(P) | 1.79 | 2.16 | 1.71 | 3.07 | 1.46 | 1.75 | 1.38 | 1.71 | 1.6 | 2.8 | 1.27 | 1.91 |
| CCC(P) | 0.61 | 0 | 0.97 | 0.89 | 0.58 | 1.37 | 0.57 | 0.57 | 0.53 | 0.4 | 0.59 | 0.99 |
| CCA(P) | 1.33 | 1.4 | 1.03 | 0 | 1.91 | 0.88 | 1.57 | 1.71 | 1.87 | 0.8 | 1.97 | 1.04 |
| CCG(P) | 0.28 | 0.44 | 0.29 | 0.04 | 0.05 | 0 | 0.48 | 0 | 0 | 0 | 0.17 | 0.06 |
| ACU(T) | 1.61 | 1.01 | 2.05 | 3.05 | 1.82 | 2.62 | 1.74 | 0.81 | 3.54 | 1.09 | 1.73 | 2.25 |
| ACC(T) | 0.8 | 0.19 | 1.06 | 0.1 | 0.76 | 0.41 | 0.77 | 0 | 0.46 | 0.57 | 0.81 | 0.65 |
| ACA(T) | 1.12 | 1.5 | 0.89 | 0.8 | 1.09 | 0.53 | 0.93 | 1.19 | 0 | 0.86 | 1.14 | 0.41 |
| ACG(T) | 0.48 | 1.31 | 0 | 0.05 | 0.34 | 0.44 | 0.56 | 2 | 0 | 1.49 | 0.32 | 0.69 |
| GCU(A) | 1.72 | 1.94 | 2.53 | 3.17 | 2.2 | 0.26 | 1.79 | 1.33 | 2.5 | 1.74 | 2.02 | 0.54 |
| GCC(A) | 0.4 | 0 | 0.47 | 0 | 0.14 | 0.09 | 0.53 | 0 | 0.11 | 0.43 | 0.29 | 0.54 |
| GCA(A) | 1.41 | 2.06 | 0.8 | 0.51 | 1.26 | 2.52 | 1.06 | 2.67 | 1.11 | 1.39 | 1.42 | 1.85 |
| GCG(A) | 0.47 | 0 | 0.2 | 0.32 | 0.4 | 1.13 | 0.62 | 0 | 0.28 | 0.43 | 0.27 | 1.07 |
| UAU(Y) | 0.87 | 0.77 | 1.34 | 1.19 | 1.54 | 1.38 | 1.03 | 1.89 | 1.37 | 1.4 | 1.51 | 1.43 |
| UAC(Y) | 1.13 | 1.23 | 0.66 | 0.81 | 0.46 | 0.62 | 0.97 | 0.11 | 0.63 | 0.6 | 0.49 | 0.57 |
| UAA | 0 | 0 | 3 | 3 | 0 | 3 | 0 | 0 | 3 | 3 | 0 | 2.5 |
| UAG | 0 | 0 | 0 | 0 | 0 | 0 | 0 | 0 | 0 | 0 | 0 | 0.3 |
| CAU(H) | 1.36 | 1.4 | 0.95 | 1.71 | 1.4 | 1.24 | 1.23 | 1.24 | 1.56 | 1.5 | 1.43 | 1.14 |
| CAC(H) | 0.64 | 0.6 | 1.05 | 0.29 | 0.6 | 0.76 | 0.77 | 0.76 | 0.44 | 0.5 | 0.57 | 0.86 |
| CAA(Q) | 1.76 | 1.33 | 1.78 | 1.68 | 1.34 | 1.78 | 1.48 | 1.11 | 1.67 | 1.8 | 1.56 | 1.75 |
| CAG(Q) | 0.24 | 0.67 | 0.22 | 0.32 | 0.66 | 0.22 | 0.52 | 0.89 | 0.33 | 0.2 | 0.44 | 0.25 |
| AAU(N) | 1.1 | 0.79 | 1.58 | 1.51 | 1.06 | 1.54 | 0.77 | 0.92 | 1.27 | 1.57 | 1.07 | 1.37 |
| AAC(N) | 0.9 | 1.21 | 0.42 | 0.49 | 0.94 | 0.46 | 1.23 | 1.08 | 0.73 | 0.43 | 0.93 | 0.63 |
| AAA(K) | 1.27 | 1.48 | 1.76 | 1.45 | 1.6 | 1.5 | 1.51 | 0.96 | 1.88 | 1.48 | 1.54 | 1.61 |
| AAG(K) | 0.73 | 0.52 | 0.24 | 0.55 | 0.4 | 0.5 | 0.49 | 1.04 | 0.12 | 0.53 | 0.46 | 0.39 |
| GAU(D) | 1.15 | 1.23 | 1.62 | 1.47 | 1.16 | 1.69 | 1.17 | 1.17 | 1.22 | 1.57 | 1.07 | 1.8 |
| GAC(D) | 0.85 | 0.77 | 0.38 | 0.53 | 0.84 | 0.31 | 0.83 | 0.83 | 0.78 | 0.43 | 0.93 | 0.2 |
| GAA(E) | 1.61 | 1.75 | 1.71 | 1.53 | 1.59 | 1.94 | 1.76 | 1 | 1.88 | 1.63 | 1.61 | 1.9 |
| GAG(E) | 0.39 | 0.25 | 0.29 | 0.47 | 0.41 | 0.06 | 0.24 | 1 | 0.12 | 0.38 | 0.39 | 0.1 |
| UGU(C) | 1.6 | 1.22 | 1.25 | 2 | 0.65 | 1.71 | 1.15 | 2 | 1.45 | 1.33 | 0.98 | 1.81 |
| UGC(C) | 0.4 | 0.78 | 0.75 | 0 | 1.35 | 0.29 | 0.85 | 0 | 0.55 | 0.67 | 1.02 | 0.19 |
| UGA | 3 | 3 | 0 | 0 | 3 | 0 | 3 | 3 | 0 | 0 | 3 | 0.2 |
| UGG(W) | 1 | 1 | 1 | 1 | 0 | 0 | 1 | 1 | 1 | 1 | 0 | 0 |
| CGU(R) | 1.44 | 2.17 | 1.43 | 1.06 | 2.41 | 2.13 | 1.42 | 2.57 | 2.33 | 0.59 | 2.43 | 1.75 |
| CGC(R) | 0.55 | 0 | 0.45 | 0.34 | 0.34 | 0.76 | 0.72 | 1.29 | 0.07 | 0 | 0.34 | 0.55 |
| CGA(R) | 1.67 | 0 | 1.22 | 1.15 | 0.56 | 0.11 | 1.47 | 0 | 1.27 | 0.12 | 0.56 | 0.69 |
| CGG(R) | 0.02 | 0.67 | 0 | 0.34 | 0.28 | 0.05 | 0.29 | 0 | 0 | 0.59 | 0.17 | 0.33 |
| AGU(S) | 0.96 | 0 | 2.03 | 2.04 | 0.22 | 1.09 | 0.85 | 0 | 1.88 | 2.09 | 0.22 | 0.97 |
| AGC(S) | 0.7 | 0.48 | 0.52 | 0.8 | 0.46 | 0.57 | 0.91 | 0.8 | 0.72 | 0.47 | 0.57 | 0.66 |
| AGA(R) | 2.14 | 1.58 | 2.58 | 1.92 | 2.16 | 2.95 | 1.93 | 2.14 | 2 | 4.71 | 2.23 | 2.65 |
| AGG(R) | 0.18 | 1.58 | 0.31 | 1.2 | 0.26 | 0 | 0.17 | 0 | 0.33 | 0 | 0.27 | 0.04 |
| GGU(G) | 1.25 | 0 | 1.93 | 0.94 | 1.26 | 2.95 | 1.24 | 1.26 | 1.75 | 1.6 | 1.61 | 2.79 |
| GGC(G) | 0.75 | 1 | 0.98 | 0 | 0.81 | 0.23 | 0.8 | 0 | 0.75 | 0.8 | 0.81 | 0.27 |
| GGA(G) | 1.89 | 2 | 1.1 | 3.06 | 1.91 | 0.79 | 1.85 | 2.74 | 1.45 | 0.8 | 1.57 | 0.77 |
| GGG(G) | 0.11 | 1 | 0 | 0 | 0.02 | 0.03 | 0.11 | 0 | 0.05 | 0.8 | 0.01 | 0.17 |

Table S2. The sequence of RBSDV and RBSDV information used in this paper

|  | accession number | host | country |
| --- | --- | --- | --- |
| RBSDV-S5 | AJ409147 | Oryza sativa | China: Zhejiang |
|  | NC003736 | Oryza sativa | China: Zhejiang |
|  | KC801047 | rice | China |
|  | HF955009 | Oryza sativa | China: Anhui |
|  | KC801046 | rice | China |
|  | AY144569 | \ | \ |
|  | KM921677 | maize | China: Jiangsu |
| SRBSDV-S5 | NC014708 | \ | China:Hainan, Dongao city |
|  | HM585275 | rice | China: Hubei |
|  | FN563987 | \ | China:Guangdong, Yangxi county |
|  | JQ034352 | rice | China: Hunan, Yiyang city |
|  | JQ692576 | rice | Viet Nam: Thua Thien Hue |
|  | HF954999 | Oryza sativa | China: Anhui |
| RBSDV-S7 | AJ297427 | rice | China: Zhejiang |
|  | NC003730 | rice | China: Zhejiang |
|  | EU111804 | rice | \ |
|  | KM921679 | maize | China: Jiangsu |
|  | AF397894 | \ | \ |
|  | AY147039 | \ | China: Henan |
|  | HF955011 | Oryza sativa | China:Anhui |
|  | KC134295 | wheat | China: Hebei Province, Baoding |
| SRBSDV-S7 | NC014710 | \ | China:Hainan, Dongao city |
|  | HF955011 | Oryza sativa | China:Anhui |
|  | JQ692578 | rice | Viet Nam: Thua Thien Hue |
|  | HM585273 | rice | China: Hubei |
|  | JQ034354 | rice | China: Hunan, Yiyang city |
|  | HM585273 | rice | China: Hubei |
| RBSDV-S9  RBSDV-S9 | KC134297 | wheat | China: Hebei Province, Baoding |
|  | AF540976 | maize and rice | China: Zhengzhou |
|  | KM921681 | maize | China: Jiangsu |
|  | AJ297430 | rice | China:Zhejiang |
|  | NC003731 | rice | China:Zhejiang |
|  | AJ291706 | maize | China:Wuhan |
|  | AF536564 | maize | China: Hubei |
|  | AY039705 | \ | China: Shandong |
|  | HF955013 | Oryza sativa | China:Anhui |
|  | AY050487 | cereal crops | China |
|  | AF459812 | cereal crops | China |
|  | HF954993 | \ | China:Anhui |
|  | HQ394211 | maize | China: Yunnan, |
| SRBSDV-S9 | HM585271 | rice | China: Hubei |
|  | JQ692580 | rice | Viet Nam: Thua Thien Hue |
|  | HM998852 | maize | China: Ji'ning, Shandong |
|  | HQ731500 | rice | Viet Nam |
|  | HF955003 | Oryza sativa | China:Anhui |
|  | NC014712 | rice | China:Hainan |
|  | EU523359 | rice | China:Hainan |
|  | JQ773428 | rice | China: Yunnan Mangshi |
